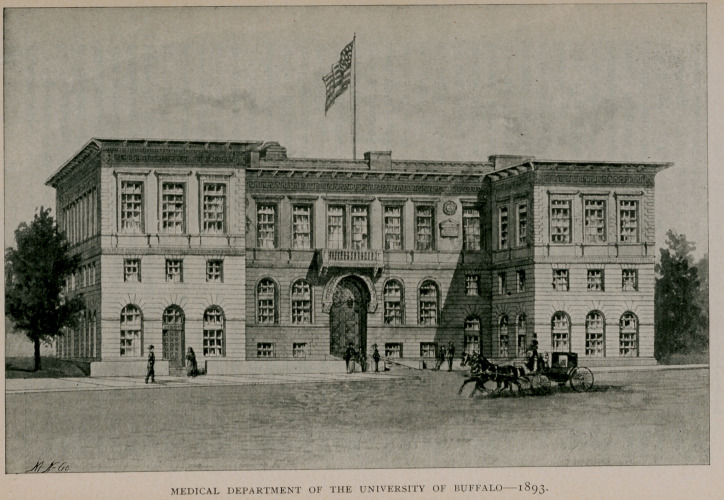# A Century of Medical History in the County of Erie.—1800–1900

**Published:** 1898-10

**Authors:** William Warren Potter

**Affiliations:** Buffalo, N. Y.


### A CENTURY OF MEDICAL HISTORY IN THE COUNTY OF ERIE.—1800-1900.

BY WILLIAM WARREN POTTER, M. D., Buffalo, N. Y.

*Pioneer Physicians—Medical Societies—Medical Colleges—Hospitals—Medical Journals—Medical Officers of the Civil War—Women Physicians—History of Homeopathy—Individual Members of the Profession.*

[*Continued from the September edition.*]

January 6, 1885, Dr. Frank Hamilton Potter was elected secretary to fill a vacancy occasioned by the resignation of Dr. Peterson. The committee on midwives reported a corrected bill to regulate their practice, and on motion it was voted to ask the president of the Medical Society of the County of Erie to call a special meeting of that body for the consideration of this bill.

Annual meeting, April 17, 1885.—Election of officers. President, Charles G. Stockton; vice-president, William Warren Potter; treasurer, F. E. L. Brecht; secretary, F. H. Potter; librarian, J. B. Samo. May 5th, the president appointed Drs. F. W. Hinkel, J. W. Putnam and E. H. Long as the standing committee on finance for the ensuing year. July 7th, on motion of Dr. Van Peyma, a committee consisting of Drs. Hopkins, Stockton, W. W. Potter, Davidson and Cary, was appointed to consider statements found in the annual report of the Buffalo Hospital of the Sisters of Charity that had been challenged. August 4th, this committee made a report which was approved by the association.

Annual meeting, April, 1886.—Election of officers: President, William Warren Potter; vice-president, J. B. Coakley; secretary, F. R. Campbell; librarian, Lucien Howe.

Annual meeting, April 5, 1887.—Election of officers: President, J. B. Coakley; vice-president, P. W. Van Peyma; secretary, C. G. Steele; treasurer, F. E. L. Brecht; librarian, Lucien Howe. On motion of Dr. A. Dagenais a building committee was appointed to solicit funds, select a site and make plans for the erection of a hall or home for the medical profession of the city. A committee was appointed by the chair to nominate the building committee, consisting of Drs. DeLancey Rochester, F. S. Crego and H. R. Hopkins. May 3d this committee reported the following names for members of the building committee: Drs. A. Dagenais, John Cronyn, Lucien Howe, William Warren Potter, Roswell Park, Conrad Diehl and Charles Cary.

Annual meeting, April, 1888.—Election of officers: President, P. W. Van Peyma; vice-president, A. A. Hubbell; secretary, W. H. Bergtold; treasurer, F. E. L. Brecht; librarian, Lucien Howe. November, 1888, Dr. Thomas Lothrop presented a memorial of Dr. F. R. Campbell, who died September 14, 1888, aged 28 years.

Annual meeting, April 2, 1889.—Election of officers: President, A. A. Hubbell; vice-president, M. B. Folwell; secretary, W. H. Bergtold; treasurer, F. E. L. Brecht; librarian, Lucien Howe. May 7, 1889, the president, Dr. Hubbell, delivered an address entitled, The Buffalo Medical and Surgical Association; retrospective and prospective. On this occasion Drs. George N. Burwell and J. B. Samo, the only surviving founders of the association, sat upon the platform by special invitation.

Annual meeting, April 1, 1890.—Election of officers: President, A. A. Hubbell; vice-president, William C. Phelps; Secretary, W. H. Bergtold; treasurer, F. E. L. Brecht; librarian, W. H. Heath.

Annual meeting, April, 1891.—Election of officers: President, William C. Phelps; vice-president, C. C. Frederick; secretary, W. Scott Renner; treasurer, F. E. L. Brecht; librarian, J. B. Coakley.

Annual meeting, April 5, 1892.—Election of officers: President, C. C. Frederick; vice-president, H. E. Hayd; secretary, William G. Ring; treasurer, F. E. L. Brecht; librarian, W. C. Callanan.

The association held a regular monthly meeting, May 10, 1892, and another June 7, 1892, both at the Hotel Iroquois. These were the last meetings of the organisation, as it had voted at a special meeting, held May 17, 1892, to unite with three other societies in forming the Buffalo Academy of Medicine. This association became the surgical section of the new organisation, and, under the compact, the officers of the association, elected April 5th, were continued as officers of the section on surgery.

### BUFFALO OBSTETRICAL SOCIETY.

On invitation, a number of physicians met at the office of Dr. William Warren Potter, January 27, 1884, to consider the propriety of organising a medical society for the consideration of subjects pertaining to obstetrics, diseases of women and pediatrics. They voted to organise the Buffalo Obstetrical Society and the first regular meeting of the new society was held February 25, 1884, at the residence of Dr. Potter. Its membership was limited to twenty-four.

The following-named officers were elected: President, William Warren Potter; vice-president, Rollin L. Banta; secretary and treasurer, George E. Fell. This was the first special medical society organised in Buffalo and it continued its work for eight years. Its proceedings were published during the greater part of this time in the Buffalo Medical Journal, and they form an interesting chapter in the medical history of the period. It was the custom of this society, as of most private medical societies, to meet at the houses of its members in rotation, and usually a collation was provided by the host after the scientific work was finished. When, in 1892, the Buffalo Academy of Medicine was organised, the obstetrical society was merged into it as a section, and its last meeting was held June 28, 1892, at the residence of Dr. Eugene A. Smith.

### Buffalo Academy of Medicine.

The propriety of creating a medical society with the foregoing title, by grouping a number of associate societies under one administration, had been discussed for some time previously, but the proposition did not take final root until May 17, 1892, when the Buffalo Academy of Medicine was founded. It was formed out of the Buffalo Medical and Surgical Association, which became the surgical section; the Obstetric Society, that became the section on obstetrics, gynecology and pediatrics; the Pathological Society, that became the section on anatomy, physiology and pathology; and the Clinical Society, that became the section on medicine, materia medica and therapeutics.

As these four several societies were already in existence, it became necessary to obtain their consent to a proposed union, which was done prior to the date before-mentioned, and the June, 1892, meetings of the several bodies were the last they held as distinct organisations. It was provided that the general meetings of the academy should be held four times a year—namely, in March, June, September and December, and that each of the constituent bodies or sections should hold regular monthly meetings. Hence there would be one meeting in each week, and these occur every Tuesday evening.

The first officers of the academy, elected June 21, 1892, were:

President, DeLancey Rochester; secretary, William C. Krauss; treasurer, Eugene A. Smith; trustees, James W. Putnam, Alphonse Dagenais and Roswell Park.

The meetings of the academy and of its several sections have been continued until the present time with a constantly increasing membership, and interesting proceedings have been varied by an occasional invitation to a nonresident, who has sometimes come from a distance to present a paper before one of the sections.

The fact that the academy has not published its proceedings with regularity makes it impossible to give as complete a sketch of this body as would be done could official or accurate data be obtained. The officers of the academy for 1898–'99: President, Dr. Roswell Park; secretary, Dr. Thomas F. Dwyer; treasurer, Dr. Charles S. Jewett; trustees, Dr. B. G. Long (three years), Dr. Marcel Hartwig (two years), Dr. DeLancey Rochester (one year).

### Private Medical Societies.

In addition to the foregoing there are also a number of private medical societies that hold regular meetings and do active and efficient work in promoting medical science. The first of these to organise was the Medical Club, that meets on alternate Wednesday evenings. The next was the Medical Union, which meets the third Tuesday in every month. The Roswell Park Medical Club is another society of this class, composed of younger physicians, that is in a flourishing condition. The Physicians' Society is still another, with its membership limited to women physicians. Perhaps the Buffalo Microscopical Society ought to be mentioned, though this is a branch of the Society of Natural Sciences. It, however, engages the attention of a number of physicians, and helps to stimulate the science of microscopy. The private societies are entertained at the houses of the members in rotation, and after the scientific work is disposed of a collation is usually provided.

### II. Medical Colleges.

Though the subject of establishing a medical college at Buffalo had been agitated previously during several years, formal steps preparatory to the application for a charter were not taken until the autumn of 1845. In the winter of 1846 authority was granted by the legislature to establish a medical school under the name and title of the Medical Department of the University of Buffalo. Hon. Nathan K. Hall, afterward postmaster-general, was then a member of the State Assembly, and it was mainly through his efforts that the charter was obtained. The first council of the university was composed of the following-named gentlemen: Ira A. Blossom, Isaac Sherman, Theodotus Burwell, James O. Putnam, Gaius B. Rich, William A. Bird, George R. Babcock, Herman A. Tucker, Joseph G. Masten, Thomas M. Foote, John D. Shepard, Millard Fillmore,

Elbridge G. Spaulding, Orson Phelps, Orsamus H. Marshall and George W. Clinton.

Public announcement of the success of the enterprise was made in the BUFFALO MEDICAL JOURNAL for September, 1846, in which it was stated that the medical department had been fully organised by creating seven professorships, to which the council of the university had made the following appointments: Chemistry and pharmacy

James Hadley; physiology and medical jurisprudence, Charles B. Coventry; general and special anatomy, James Webster; pathology and materia medica, Charles Alfred Lee; principles and practice of

HON. JAMES O. PUTNAM, CHANCELLOR OF U. OF B.

surgery, Frank Hastings Hamilton; obstetrics and diseases of women and children, James Platt White; principles and practice of medicine and clinical medicine, Austin Flint. Corydon L. Ford was appointed demonstrator of anatomy. The first five teachers above named were holding similar chairs in Geneva Medical College, an institution that soon afterward was discontinued. Dr. Hamilton removed to Buffalo in 1845, Dr. Webster retained his residence in Rochester, Dr. Coventry his at Utica, while James Hadley's son George delivered the chemistry lectures from the beginning, and was soon appointed to the chair.

The chief promoters of the college enterprise were Drs. White, Flint and Hamilton, who were ably seconded by Mr. O. H. Marshall and several other prominent citizens. Millard Fillmore, afterward president of the United States, was the first chancellor of the university, an office which he continued to fill until his death, March 8, 1874. He was succeeded by Orsamus H. Marshall, and he by E. Carlton Sprague, who in turn was followed by James O. Putnam, the present incumbent, who has been a member of the council from the outset. This was the beginning of the first permanently successful effort to establish in Buffalo an educational institution above the grade of common schools.

FIRST BUFFALO MEDICAL COLLEGE, WASHINGTON AND SENECA STREETS.

The structure occupied by the college during its first three academic years, known as the first Baptist Church, then stood on the corner of Seneca and Washington streets, the site of the present post-office building. The first course of medical lectures opened February 24, 1847, with an attendance of sixty-six registered students, one of whom was Mr. L. G. Sellstedt, the distinguished artist of Buffalo, who took a special course. The first commencement was held at the First Presbyterian church, June 16, 1847, at which Hon. Millard Fillmore, chancellor, after a brief address, conferred the degree of doctor of medicine upon seventeen gentlemen, whose names were as follows: George Abbott, M. H. Andrews, H. W. Barrett, Z. A. Blake, John P. Dudley, Sidney A. Foss, H. D. Garvin, John Hardy, James E. King, S. G. Rogers, Wells Taber and J. A. Whiting. Of these Dr. Abbott is still living and engaged in the practice of his profession at Hamburg.

The address to the graduates was delivered by the dean of the medical faculty, Prof. Frank Hastings Hamilton, the exercises were interspersed with music, and closed with a benediction by the Rev. Mr. Schuyler of St. John's church. These were the first literary exercises of the kind ever witnessed in Buffalo, and they were attended by a large concourse of prominent citizens.

At the second annual commencement, June 14, 1848, there were thirty-two graduates out of a total attendance of ninety-five students. In the absence of the chancellor, Dr. Thomas M. Foote, the vice-chancellor, conferred the degrees, and Professor Austin Flint delivered the address to the graduating class. Among those to receive medical degrees on this occasion was Dr. C. C. Wyckoff, who is at present in active professional practice in Buffalo.

The church structure referred to was used by the college during its first three academic years. By this time, however, the necessity presented itself for increased accommodations, which culminated in locating the school in a building of its own. Public-spirited citizens were invited to contribute to the enterprise through the medium of a subscription list that was circulated. This was headed by A. D. Patchen, who subscribed $500; next came Jesse Ketchum, who gave $600, the largest single donation, and then followed in their order the names of Albert H. Tracy, George W. Tifft, Eldridge G. Spaulding and Jabez Goodell, who each gave $200. There were eighty citizens who subscribed $100 each, and the remainder was raised in sums of $60 and $40, until the aggregate subscription reached $12,000. The State appropriated $2,000, which made a sufficient amount to justify the commencement of the construction of the new edifice. Meanwhile land was purchased at the corner of Main and Virginia streets, a location then quite outside of the city, and the construction of the building was begun under the most inspiriting auspices. It was completed in season for the fourth lecture course, 1849–50, at a cost of about $15,000. It would be interesting to trace the history of the college from this time forward, but it must suffice to record a few of the important events that have occurred during its existence.

It was during its fourth year that Dr. White introduced demonstrative or clinical midwifery into the college curriculum, a method of teaching that had already been established in Europe, but had not been attempted before in this country. Part of the plan was as follows: A woman, two weeks before confinement, entered the janitor's apartments, where she was boarded and cared for by the janitor's

SECOND BUFFALO MEDICAL COLLEGE, MAIN AND VIRGINIA STREETS.

wife. After labor began the graduating class, twenty-two in number, assembled in an adjoining room, and one by one under the supervision of Dr. White were admitted to the confinement room and were permitted to make vaginal examinations during the progress of labor. On the termination of the second stage all were assembled in the lying-in room and permitted to witness the passage of the head over the perineum, as well as the method employed to support the latter. This was all; there was no undue exposure of the woman and she made a rapid convalescence; yet seldom has an event occurred that so completely shook the foundations of society in any city as did this.

Newspapers commented upon it, doctors denounced it as immoral, and a suit for libel followed. A scathing critique signed "L" appeared in one of the daily newspapers, reflecting so intemperately upon Dr. White's course that he promptly brought suit for libel against Dr. Horatio N. Loomis, the supposed author of the article; for it was known that Dr. Loomis had expressed himself verbally in opposition to this method of teaching. A trial ensued, lasting four days, able counsel appeared on both sides, two stenographers were employed by the complainant (this was before the days of court stenographers) and a full report was made and published to the world. Much stress had been laid by the counsel for the defendant upon the fact that "public opinion" placed the stamp of its emphatic disapproval upon the course of Dr. White. Mr. Justice Mullett, who presided at the trial, swept all such fallacies from the jury box in a terse and able charge which reached a climax of haughty eloquence in the following paragraph:

"Public opinion has not in Christendom been deemed a very safe agent in the administration of justice since it profaned the judgment seat and insulted Heaven by the cry of crucify Him! crucify Him!! Pilate, weak and time serving, disobeyed the dictates of his own conscience and followed the popular outcry which he mistook for public opinion. But the sacred history of that awful tragedy informs us that the chief priests and elders persuaded the multitude."

Dr. Loomis was acquitted, for it was proved that another had written the libel, but Dr. White was vindicated. His name will be handed down during all time as the first in America to attempt the clinical teaching of midwifery. Dr. White continued his work as a teacher from 1846 to 1881, during which time he inaugurated many methods of improvement in his specialty and successfully performed many difficult operations in abdominal and pelvic surgery. He was in advance of his time in many respects, and left a name that will always be conspicuous for having contributed much to the advancement of the science of medicine. He devised many ingenious instruments, and his obstetric forceps is well known throughout the land. Since his death, which ocurred September 28, 1881, Dr. Matthew D. Mann, who was soon afterward appointed to the chair made vacant, has continued to teach obstetrics and gynecology in the university.

Dr. Frank Hastings Hamilton, who was the first teacher of surgery, held the chair until his removal to New York in 1860. During the fifteen years of his residence in Buffalo he did much original work that served to establish him among the first surgeons in the country and he was ever afterward recognised as such. He published fracture tables and introduced new methods in the treatment of fractures, which laid the foundation for his classic treatise on fractures and dislocations that has been translated into several foreign languages.

Dr. Hamilton possessed special qualifications as a teacher and it is doubtful if his superior has ever been found in this country either in the amphitheatre or at the bedside.

After Dr. Hamilton's removal to New York, Dr. E. M. Moore, of Rochester, who had been teaching surgical pathology in the college for some years, was appointed to the chair of surgery. Professor Moore was an original thinker and an attractive teacher as well as a resourceful surgeon. He continued to occupy this chair until 1883 when he resigned on account of advancing years. Dr. Roswell Park, of Chicago, was appointed to fill the vacancy thus created and has been teaching in that capacity until the present time.

In 1867 the chair of special surgery was created and Dr. Julius F. Miner was invited to fill it. It was not long before Professor Miner developed popularity as a teacher and skill as a surgeon. He continued this work until failing health in 1884 compelled him to resign. In 1869, Dr. Miner demonstrated the feasibility and propriety of applying the principles of enucleation in the removal of ovarian tumors. This ingenious and scientific suggestion was adopted throughout the world whenever applicable and it made Professor Miner's name famous in literature.

In 1851, Dr. Coventry resigned the chair of physiology and Dr. John C. Dalton, Jr., was appointed to succeed him. Dalton had been a pupil of the great French physiologist, Bernard, and he at once instituted the methods of the latter in illustrating his lectures by vivisections before the class. This was the first time the method had been adopted in this country—a system of teaching which has since gained universal application. Dr. Dalton held this chair until 1858, when he went to New York and continued his work in that city. He became the author of a text-book on physiology that was almost universally adopted. He died at New York, February 12, 1889, aged 64 years, after having obtained conspicuous prominence as an author and teacher of physiology.

Dr. Austin Flint one of the founders of the college, taught the practice of medicine from 1846 to 1853. In the latter year he was invited to Louisville and subsequently to New Orleans, in both of which cities he taught internal medicine for several years. Finally, he went to New York and occupied the chair of practice of medicine at the Bellevue Medical College until his death, March 13, 1886. He was a recognised authority in diseases of the chest and he reduced physical exploration to a scientific exactitude that had not been heard of before his time. His ear was so finely attuned to rhythmical sounds that he was enabled to detect minute chest râles that were not easily differentiated by others, and to give them a fixed and definite significance in the pathology of pulmonary diseases. He was a voluminous writer, his works have been recognised everywhere as standard authority, and have in some instances come to be regarded as classic. He specially distinguished himself in establishing the true nature of the infection of typhoid fever as early as 1843. A well at North Boston, N. Y., became poisoned by the excreta of a typhoid patient brought from Massachusetts. Twenty-one cases occurred in families living within a few rods of the well, from which they obtained their water supply, of whom seven died. Dr. Flint visited the scene, diagnosticated and traced an infectious disease, then unknown in this region, from New England to that obscure hamlet, distinctly established its contagion, and pointed out its source. The published report became a classic in medical literature that will always be referred to, and it formed the basis of a series of essays afterward published by Prof. Flint on the subject of typhoid fever.

In 1853, Dr. Thomas F. Rochester, a native of the city that bears his name, who had lately been serving in Bellevue Hospital, was appointed professor of practice to fill the vacancy caused by Dr. Flint's removal. An incident in his life deserves to be recorded in this place. He was one of twelve young men who entered the hospital at Bellevue as unpaid assistants. Soon afterward a fearful pestilence invaded the wards, and at the close of the year seven of the little band had died while performing the duty named. They rode not down to the valley of death in a magnificent charge with banners and trumpets, like the historic 600 at Balaklava, but they went down to a pestilential battlefield just as consciously, just as heroically. No mausoleum, no obelisk, no monumental bronze marks their resting place to perpetuate their deeds. Only on a mural tablet at Bellevue may be read the record of this great martyrdom. The facts and circumstances are recorded here as received from an intimate friend of Dr. Rochester who was familiar with the circumstances. Dr. Rochester did much to advance the science of medicine, and was one of the most conscientious and progressive teachers in the college. He continued to perform his labors until within a few months of his death, which occurred May 24, 1887, when he was 63 years of age. He was succeeded by Dr. Charles G. Stockton, who still occupies the chair of medicine.

Dr. Corydon L. Ford, who afterward attained conspicuous eminence as an anatomist, resigned the demonstratorship of anatomy in 1853, and Dr. Sanford B. Hunt, of Mendon, N. Y., was appointed to fill the vacancy. The following year Dr. Hunt was advanced to the professorship of anatomy, which he held until 1858. He was a man of science and made anatomy an interesting subject to his pupils. Instead of the usual dry methods of teaching, he adopted those that directed attention and fixed the memory through the novelty of surrounding it with more than ordinary interest. He resigned the chair to engage in editorial and educational work, and was elected superintendent of public schools.

MILTON GROSVENOR POTTER, M. D.

Dr. Sandford Eastman, an alumnus of the college, was appointed to fill the vacancy and held the chair until his death, January 8, 1874. Dr. Eastman was one of the most popular teachers in the college, one who commanded the respect alike of all his colleagues and his pupils, as well as the love and esteem of a large clientèle that mourned his decease as a loss of inestimable moment.

Dr. Milton Grosvenor Potter, also an alumnus of the college, was chosen to fill the vacancy. He, too, was an interesting and forceful teacher who obtained at once the respect and love of his pupils as well as the most distinguished consideration of his colleagues. He

MEDICAL DEPARTMENT OF THE UNIVERSITY OF BUFFALO—1893.

died January 28, 1878, lamented by a large circle of friends and acquaintances.

The chair of materia medica was nominally held by Professor Lee from the beginning down to 1870. The lectures, however, were delivered during his unavoidable absence by Professor Theophilus Mack from 1857 to 1860, and by Professor Joshua R. Lothrop from 1860 to 1864. When Dr. Lee resigned in 1870, Professor H. N. Eastman was appointed to the chair and he was succeeded in 1873 by Dr. E. V. Stoddard, of Rochester, who held it until 1888, when Dr. Charles Cary, who upon Dr. Potter's death had been appointed professor of anatomy, was transferred at his own request to fill the vacancy occasioned by Professor Stoddard's resignation. Professor Cary is still engaged in teaching materia medica and therapeutics at the college.

The following-named gentlemen have served as demonstrators of anatomy from 1846 to the present writing, viz.: Corydon L. Ford, Sanford B. Hunt, John Boardman, Benjamin H. Lemon, Hugh B. Van Deventer, S. W. Wetmore, M. B. Folwell and William C. Phelps. Dr. Phelps still continues as demonstrator and has also been made assistant professor of anatomy. A few years ago the college building, at the corner of Main and Virginia streets, became unsuited to modern methods in medical teaching, as well as too limited in its capacity to accommodate the increasing attendance of students. Its anatomical rooms were inadequate; its laboratories too restricted; its amphitheaters were too small, and in short the methods of 1890 had outgrown those of 1850. Though it was a comely structure and the first building erected on the Holland Purchase for collegiate instruction since the soil on which it stands was relinquished by the Senecas, it has ceased to be occupied and is fast falling into decay. Since the foregoing was written it has been razed and a new structure has been built upon the site.

Ground was obtained on High street in the vicinity of the General Hospital, and the construction of a new building put under way in 1892. The present college edifice was opened March 5, 1893, with public ceremonies befitting the occasion. This superb building is admirably adapted to the purposes of medical instruction and it fittingly bespeaks the energy and sagacity of its projectors. The remaining part the college is to play in history concerns the immediate present and the future which is yet to be written.

We may very fittingly close this chapter by recording the names of the successors of the original seven teachers. They who are now in office are as follows: Charles Cary, professor of materia medica, therapeutics and clinical medicine; Matthew D. Mann, dean, professor of obstetrics and gynecology; Roswell Park, professor of the principles and practice of surgery and clinical surgery; Julius Pohlman, professor of physiology; Charles G. Stockton, professor of the principles and practice of medicine and clinical medicine; John Parmenter, secretary, professor of anatomy and adjunct professor of clinical surgery; Herbert M. Hill, professor of chemistry, toxicology and physics.

In addition to these there are seven adjunct professors, seventeen professors of special departments and eleven instructors, besides a number of clinical instructors and students' assistants. This array of teachers contrasted with the original seven indicates the progress in medical instruction during the last fifty years. As a further evidence of progress it may be mentioned that the following departments have been erected in the University since the creation of the medical department in 1846—namely, the department of pharmacy, established in 1886; the department of law, established in 1887; the department of dentistry, established in 1892, and the school of pedagogy, established in 1895. It is, therefore, a university in fact as well as in law.

Since the foregoing paragraph was penned Niagara University Medical College has been consolidated with the Buffalo School, thus further augmenting the teaching corps.

(*Continued next month.*)

## Figures and Tables

**Figure f1:**
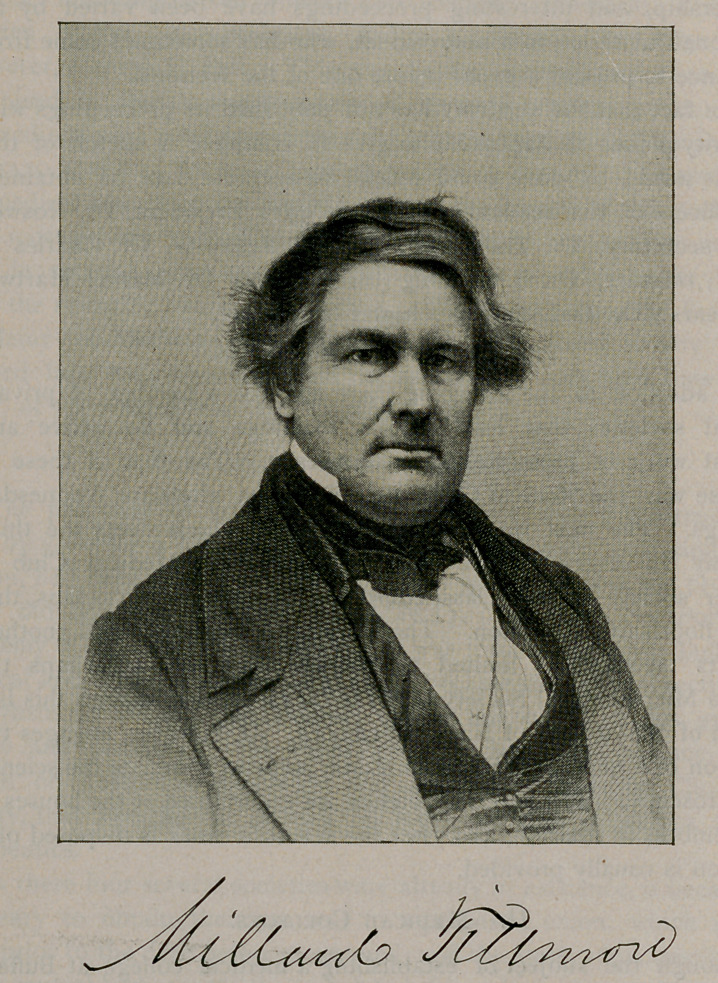


**Figure f2:**
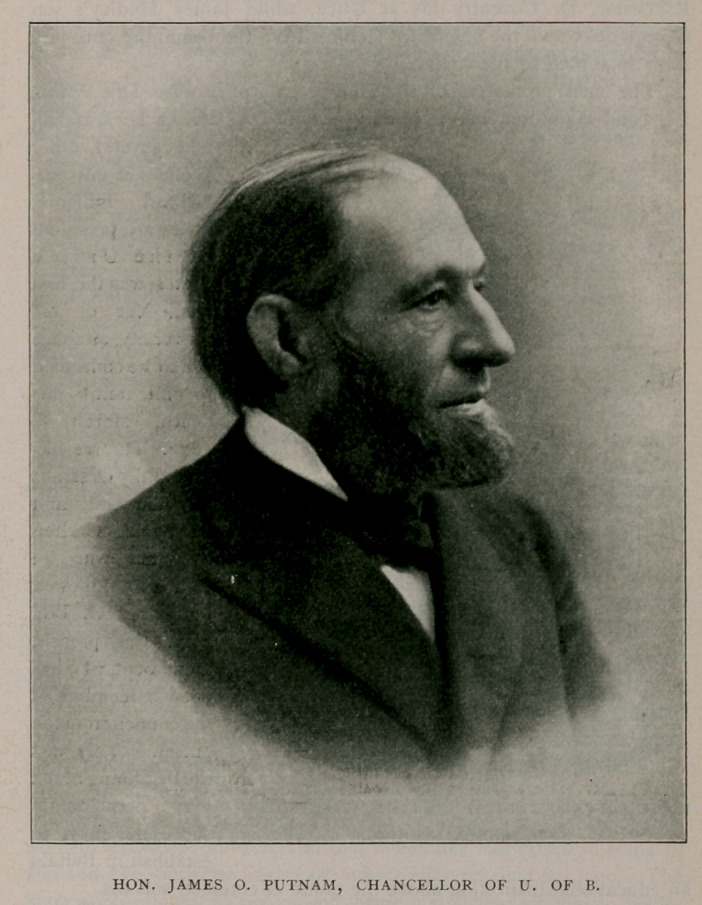


**Figure f3:**
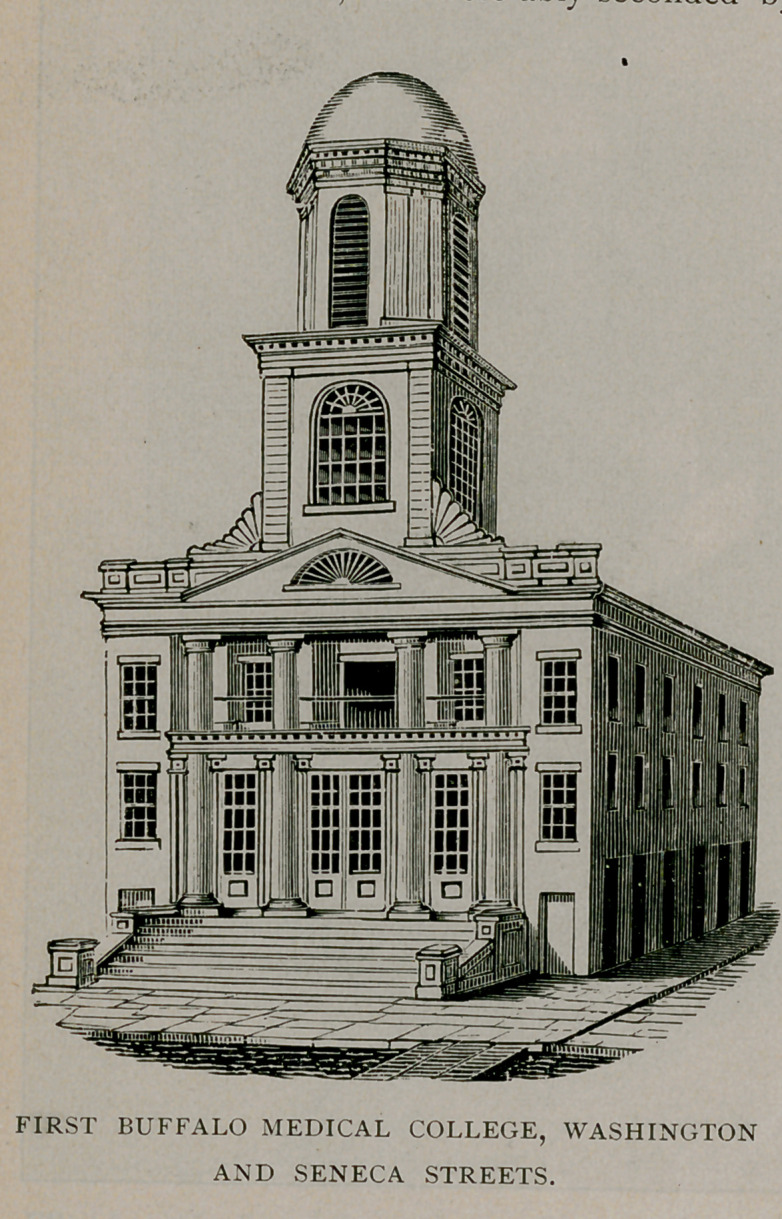


**Figure f4:**
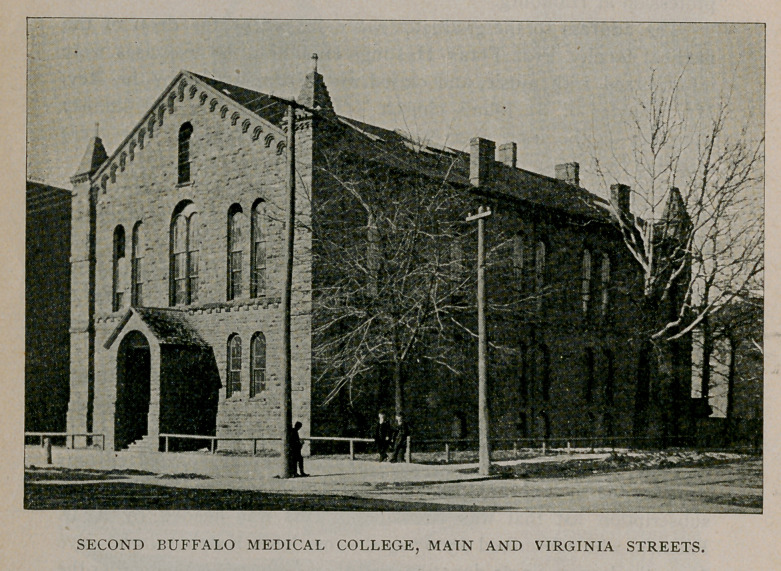


**Figure f5:**
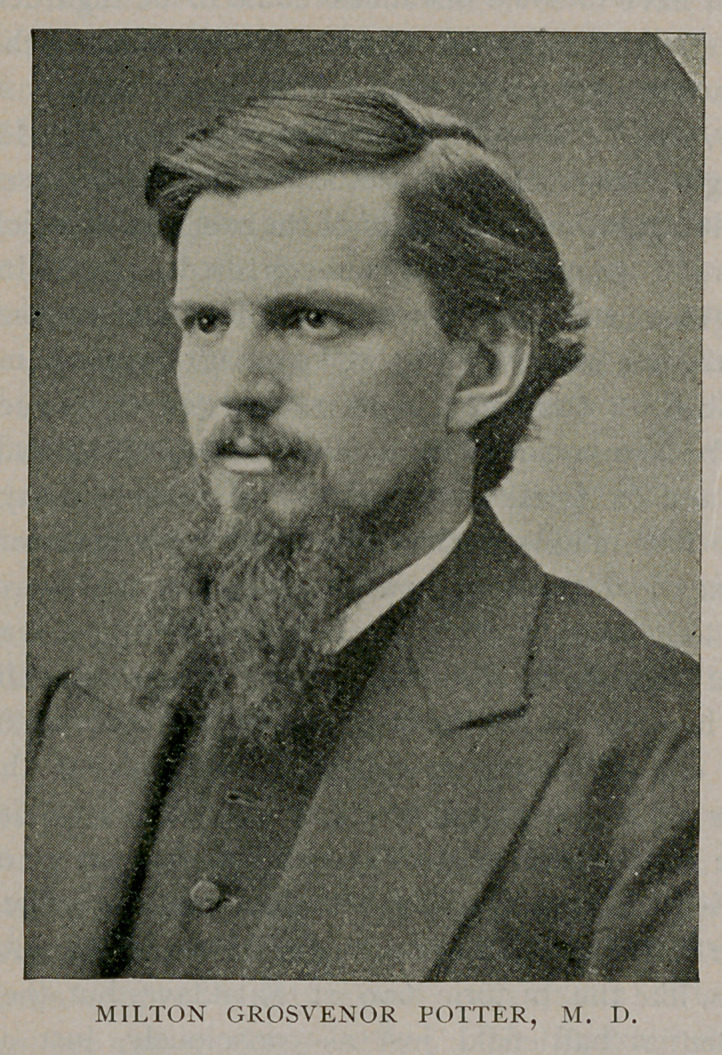


**Figure f6:**